# Local sparsity enhanced compressed sensing magnetic resonance imaging in uniform discrete curvelet domain

**DOI:** 10.1186/s12880-015-0065-0

**Published:** 2015-08-08

**Authors:** Bingxin Yang, Min Yuan, Yide Ma, Jiuwen Zhang, Kun Zhan

**Affiliations:** School of Information Science & Engineering, Lanzhou University, Tianshui South Road No.222, Lanzhou, 730000 China

**Keywords:** Compressed sensing, Magnetic resonance imaging, Uniform discrete curvelet transform, Dictionary learning, Augmented Lagrangian

## Abstract

**Background:**

Compressed sensing(CS) has been well applied to speed up imaging by exploring image sparsity over predefined basis functions or learnt dictionary. Firstly, the sparse representation is generally obtained in a single transform domain by using wavelet-like methods, which cannot produce optimal sparsity considering sparsity, data adaptivity and computational complexity. Secondly, most state-of-the-art reconstruction models seldom consider composite regularization upon the various structural features of images and transform coefficients sub-bands. Therefore, these two points lead to high sampling rates for reconstructing high-quality images.

**Methods:**

In this paper, an efficient composite sparsity structure is proposed. It learns adaptive dictionary from lowpass uniform discrete curvelet transform sub-band coefficients patches. Consistent with the sparsity structure, a novel composite regularization reconstruction model is developed to improve reconstruction results from highly undersampled *k*-space data. It is established via minimizing spatial image and lowpass sub-band coefficients total variation regularization, transform sub-bands coefficients *l*_1_ sparse regularization and constraining *k*-space measurements fidelity. A new augmented Lagrangian method is then introduced to optimize the reconstruction model. It updates representation coefficients of lowpass sub-band coefficients over dictionary, transform sub-bands coefficients and *k*-space measurements upon the ideas of constrained split augmented Lagrangian shrinkage algorithm.

**Results:**

Experimental results on in vivo data show that the proposed method obtains high-quality reconstructed images. The reconstructed images exhibit the least aliasing artifacts and reconstruction error among current CS MRI methods.

**Conclusions:**

The proposed sparsity structure can fit and provide hierarchical sparsity for magnetic resonance images simultaneously, bridging the gap between predefined sparse representation methods and explicit dictionary. The new augmented Lagrangian method provides solutions fully complying to the composite regularization reconstruction model with fast convergence speed.

## Background

Compressed sensing(CS) was first presented in the literature of Information Theory as an abstract mathematical idea [1, 2]. The fundamental idea behind CS is: rather than first sampling at a high rate and then compressing the sampled data, directly sensing the data in a compressed form (at a lower sampling rate) is preferred. CS points out that a signal can be recovered exactly from a small set of random, linear and nonadaptive measurements if it has a sparse representation. Suppose $\textbf {x}\in \mathbb {C}^{p}$ denotes the unknown compressible (*N*-sparse) signal to be reconstructed. ***Ψ*** denotes a tight frame sparse transform matrix. Then **x** can be sparsely represented as ***α***=***Ψ*** **x**, where ∥***α***∥_0_=*N*(*N*≪*p*). It is possible to measure a relatively small number of “random" linear combinations of signal (much smaller than the number of signal samples nominally defining it) allowing accurate reconstruction, which is comparable to that attainable with direct knowledge of the *N* most important coefficients. The measurement process is denoted as **y**=***Φ*** **x**, where $\boldsymbol {\Phi }\in \mathbb {C}^{m\times p} \left (m\ll p\right)$ denotes measurement matrix irrelevant to the sparse transform basis. Thus

(1) $$ \textbf{y}=\boldsymbol{\Phi}\textbf{x}=\boldsymbol{\Phi}\boldsymbol{\Psi}^{-1}\boldsymbol{\alpha}  $$

in which ***Φ*** ***Ψ*** ^−1^ is termed as the sensing matrix. The sensing matrix should satisfy three properties including the null space property, restricted isometry property and bounded coherence [3]. Given measurements **y** and the sensing matrix, the reconstruction problem turns out to be an optimization problem of the form

(2) $$ \arg\min_{\textbf{x}, \hat{\boldsymbol{\alpha}}}\left\|\hat{\boldsymbol{\alpha}}\right\|_{0} \ \ \text{s.t.} \ \boldsymbol{\Phi}\boldsymbol{\Psi}^{-1}\hat{\boldsymbol{\alpha}}=\textbf{y}  $$

(2) can be solved by various nonlinear reconstruction approaches.

In magnetic resonance imaging(MRI), the sampled combinations are simply individual Fourier coefficients (*k*-space samples). MRI is a relatively slow imaging modality at a limited data acquisition speed. Undersampling *k*-space allows speeding up imaging but introduces aliasing in the reconstructed magnetic resonance(MR) images simultaneously, because it violates the Nyquist sampling theorem. Compared with that by the sinc function interpolation using sampled data restricted by Nyquist sampling theorem, CS enables MR image reconstruction with little or no visual information loss from randomly undersampled *k*-space measurements. Hence, it is natural to introduce CS into undersampled MRI. The emerging method to reduce MRI scanning time via CS is termed CS MRI [4, 5]. Three requirements for successful CS MRI are: the MR image can be sparsely represented (compressible); the aliasing artifacts brought by *k*-space undersampling are incoherent (noise like) in the transform domain; then CS solves the general reconstruction formulation using nonlinear method by constraining both sparsity and *k*-space measurements consistency. In CS MRI, incoherent random, radial and spiral trajectories [4, 6, 7], etc, are used to acquire measurements from *k*-space.

Sparsity is of vital importance for reducing artifacts in CS MRI reconstruction. The generally used sparse representation methods are spatial finite difference [4, 8, 9], discrete wavelet transform(DWT) [4, 8, 9], sharp frequency localization contourlet(SFLCT) [10, 11], discrete curvelet transform using fast algorithm(FDCT) [12–14], discrete shearlet transform(DST) [15, 16], sparsity along temporal axis for dynamic cardiac imaging [17–19] and the combination of some of these transforms [4, 20, 21]. Dictionary has also been introduced for sparse representation and adaptive data fitting [22, 23] and it is learnt from intermediate reconstructed or fully sampled images. Furthermore, double sparsity model has been proposed likewise. It combines adaptive dictionary learning(DL) with predefined sparse priors for signal flexible representations, stability under noise and reducing overfitting [24, 25]. Besides, nonlocal processing [26] methods have been explored as well based on the similarity of image patches [27–30] and sparsity originated from this similarity [31, 32]. Established on the above sparse representation approaches, various CS MRI methods have been presented for handling the ill-posed linear inverse problem, including convex, nonsmooth sparse regularization (*l*_1_ and total variation) based LDP [4], TVCMRI [33], iterative thresholding CS MRI based on SFLCT [11], FCSA [21], NLTV-MRI upon nonlocal total variation(TV) [9], reconstruction under wavelet structured sparsity(WaTMRI) studied in [34], reconstruction via using DL [35–37] and PANO [32] by using variable splitting(VS) and quadratic penalty reconstruction technique [38] incorporated with patch-based nonlocal operator, etc. Besides, a novel individual MRI reconstruction framework of low-rank modeling for local *k*-space neighborhoods(LORAKS) [39] was also proposed. LORAKS generated support and phase constraints in a fundamentally different way from more direct regularized methods [4, 40]. Among these reconstruction methods, DWT based MRI reconstruction gave rise to feature loss and edge blur with numerous aliasing in reconstructed images. WaTMRI provided new thought for CS MRI by making full use of the coefficients structural relationship. PANO was recently proposed to sparsify MR images by using the similarity of image patches, achieved considerably lower reconstruction error and allowed us to establish a general formulation to constrain the sparsity of similar patches and data consistency. The availability of guide image and how the gridding process affect PANO imaging with nonCartesian sampling remain to be carefully analyzed. Besides, LORAKS provided very flexible implementation and was easily incorporated with other constraints. Furthermore, 3D dynamic parallel imaging has also been presented and was of great significance for practical MRI applications. 3D dynamic parallel imaging was generally established on TV and sparsity along temporal axis [17–19] and structured low-rank matrix completion [41–45].

In this paper, a novel composite sparsity structure is developed, which is inspired by double sparsity model. In this composite sparsity structure, uniform discrete curvelet transform(UDCT) [46] decomposes MR images hierarchically into one lowpass sub-band and several other highpass sub-bands. Then an adaptive dictionary is learnt from the hardly sparse lowpass sub-band coefficients patches. This adaptive DL allows a smaller amount of calculation with little (or no) decrease of efficiency compared with the double sparsity model. UDCT has quite similar properties to those of wrapping-based FDCT, such as *C*(logN)^3^ *N* ^−2^ mean square error(MSE) decay rate for *C* ^2^-singularities signal with *N* most important transform coefficients in the curvelet expansion, tight frame property, highly directional sensitivity and anisotropy in the sense that they both employ alias free subsampling in frequency domain. Additionally, UDCT possesses some specialities making it superior over FDCT in CS MRI applications, such as a smaller redundancy of 4 and clear coefficients parent-children relationship. The goal of the proposed composite sparsity structure is to capture various directional features of images hierarchically, provide more flexible and sparse representations for lowpass sub-band adaptively, and reduce overfitting and computational complexity simultaneously. Consistent with this composite sparsity structure, one reconstruction model is provided. It involves minimizing UDCT sub-bands coefficients *l*_1_ regularization, image and lowpass sub-band coefficients TV penalty and constraining *k*-space measurements fidelity. Then a new fast convergent augmented Lagrangian(AL) reconstruction method is presented to solve the reconstruction model. It is established on the constrained split augmented Lagrangian shrinkage algorithm(C-SALSA) [47], translates the original formulation into another constrained one via VS and then solves the constrained one by using the variant of ADMM [48, 49](ADMM-2 [47]). The ADMM-2 resulting from our reconstruction problem involves quadratic problem (which can be solved exactly in closed form), *l*_1_ regularization, a shrinkage operation and an orthogonal projection on a ball.

The remainder of this paper is organized as follows. Section “Methods” depicts the prior work, the proposed CS MRI method including the composite sparsity structure and corresponding reconstruction model, and its validity in detail. In section “Results and discussions” some reconstruction results are exhibited for the proposed method and current CS MRI techniques. The ability in handling noise, convergence speed and influences of the proposed reconstruction model parameters fluctuation are analyzed, etc. Finally, conclusions and future work are explicit in section Conclusions and future work.

## Methods

### Compressed sensing MRI prior art

In CS MRI, $\textbf {x}\in \mathbb {C}^{p}$ denotes the vector form of the 2D MR image to be reconstructed and **y**=**F**_*u*_ **x** denotes the sensing procedure in *k*-space, where $\textbf {F}_{u}\in \mathbb {C}^{m\times p}\left (m \ll p\right)$ means undersampled Fourier Encoding matrix and $\textbf {y}\in \mathbb {C}^{m}$ represents *k*-space measurements. Assume ***Ψ*** represents an analytical sparse transform operator or the inverse of a set of signals learnt from image patches, the sparse representation is defined as ***α***=***Ψ*** **x**. Reconstructing the unknown MR image **x** from measurements **y** by using CS is to solve the constrained CS MRI optimization problem (3)

(3) $$ \min_{\textbf{x}} \left\|\boldsymbol{\Psi} \textbf{x}\right\|_{1} \ \ \text{s.t.} \ \left\|\textbf{F}_{u} \textbf{x}-\textbf{y}\right\|^{2}_{2} \leq \boldsymbol{\varepsilon}  $$

where $\boldsymbol {\varepsilon }\in \mathbb {C}^{m}$ represents the allowed noise vector in reconstructed image, *l*_1_ regularization enforces sparsity of ***Ψ*** **x** and error constraint fits the reconstruction to the sampled *k*-space measurements. Finite difference referred to as TV is typically added into (3) for noise reduction and spatial homogeneity enhancement. Then the formulation is

(4) $$ \min_{\textbf{x}} \left\|\boldsymbol{\Psi} \textbf{x}\right\|_{1}+ \lambda TV\left(\textbf{x}\right) \ \ \text{s.t.} \ \left\|\textbf{F}_{u} \textbf{x}-\textbf{y}\right\|^{2}_{2} \leq \boldsymbol{\varepsilon}  $$

where *λ*>0 denotes the weight of TV regularization. This becomes problem formulation settled by classical LDP when ***Ψ*** represents DWT. Besides, most state-of-the-art AL based reconstruction methods consider one unconstrained problem rather than (4), such as TVCMRI, FCSA, iterative thresholding CS MRI based on SFLCT and SALSA [50], etc

(5) $$ \min_{\textbf{x}} \lambda_{1}\left\|\boldsymbol{\Psi} \textbf{x}\right\|_{1}+ \lambda_{2} TV\left(\textbf{x}\right)+\frac{1}{2}\left\|\textbf{F}_{u} \textbf{x}-\textbf{y}\right\|^{2}_{2}  $$

Additionally, reconstruction based on explicit DL from image patches has been explored

(6) $$ \begin{aligned} &\min_{\textbf{x},\textbf{D},\boldsymbol{\Gamma}} \sum\limits_{ij}\left\|\textbf{R}_{ij}\textbf{x}-\textbf{D}\boldsymbol{\alpha}_{ij}\right\|^{2}_{2}+ \lambda \left\|\textbf{F}_{u} \textbf{x}-\textbf{y}\right\|^{2}_{2} \ \text{s.t.} \ \left\|\boldsymbol{\alpha}_{ij}\right\|_{0} \\ &\quad\leq T_{0}, \forall i,j \end{aligned}  $$

(6) is the formulation settled by DLMRI integrating DL with image reconstruction simultaneously (refer to [35]), where matrix $\textbf {R}_{\textit {ij}}\in \mathbb {C}^{n \times p}$ is an operator that extracts maximum overlapped patch **x**_*ij*_ as a column vector from **x**, denoted as **x**_*ij*_=**R**_*ij*_ **x**. $\sum _{\textit {ij}}\textbf {R}_{\textit {ij}}^{T} \textbf {R}_{\textit {ij}}\in \mathbb {C}^{p \times p}$ represents a diagonal matrix, in which the diagonal elements correspond to pixel locations of **x**. ***Γ*** is sparse representation set {***α***_*ij*_}_*ij*_ of all training patches of image. *T*_0_ denotes sparse threshold and **D** the explicit dictionary. Here DL problem is depicted in detail as a foundation of the proposed sparsity structure. The DL problem is

(7) $$ \min_{\textbf{D}, \boldsymbol{\Gamma}} \sum\limits_{ij}\left\|\textbf{R}_{ij}\textbf{x}-\textbf{D}\boldsymbol{\alpha}_{ij}\right\|^{2}_{2} \ \ \text{s.t.} \ \left\|\boldsymbol{\alpha}_{ij}\right\|_{0} \leq T_{0}, \forall i,j  $$

Formulation (7) is nonconvex and NP-hard [51, 52] because it comes down to sparse encoding problem for fixed **D** and **x**. K-SVD [53, 54] is a simple but efficient approach to attack (7). It simultaneously implements dictionary update step where each atom of **D** renews sequentially and corresponding sparse encoding for image patches that currently use it. The dictionary atom update involves computing *K* singular value decompositions(SVDs), once for each atom.

### Proposed method

**Fig. 1:**
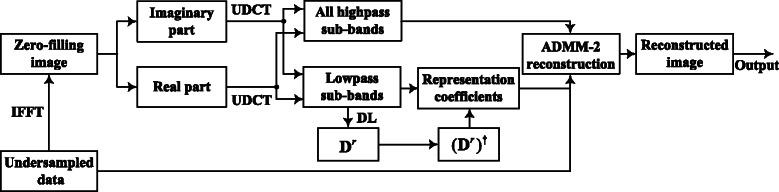
Framework of local sparsity enhanced CS MRI reconstruction

In this section, a composite regularization CS MRI method established on a novel composite sparsity structure is presented. In the sparsity structure, UDCT decomposes spatial image into one lowpass sub-band and several highpass sub-bands. Patch-based dictionary is learnt from the lowpass sub-band coefficients patches via Sparse K-SVD [25]. Then a novel composite regularization reconstruction model is thereby established and solved via VS and ADMM-2. The reconstruction model involves spatial image and transform coefficients regularization and *k*-space data fitting. The framework in Fig. 1 shows clearly the implementation process of the proposed method, in which the unknown MR image **x** is initialized with a zero-filling reconstructed image via direct inverse Fourier transform to *k*-space measurements, denoted as $\textbf {x}_{0}=\textbf {F}^{H}_{u}\textbf {y}$. UDCT decomposes both the real and imaginary parts of **x**_0_ into *S* levels, each level possessing 2*κ*_*s*_ directional sub-bands. The real and imaginary parts of complex-valued MR image are handled separately because UDCT can only perfectly deal with real-valued data. Take the real part of zero-filling reconstructed image for example, the lowpass UDCT sub-band is divided into maximum overlapped patches (for dividing method, refer to [35]) as training database for DL to enhance its sparsity. The obtained dictionary **D** ^*r*^ (*r*=1(0) denotes result over real (imaginary) part) is the result of Sparse K-SVD to the training database. The sparse encodings set are referred to as the double sparse coefficients for all training lowpass UDCT sub-band patches over learnt **D** ^*r*^. For imaginary part, the same procedure is implemented. Let **x**_0_ be the initial intermediate image and (**D** ^*r*^)^*†*^ the pseudo-inverse of **D** ^*r*^. The reconstruction step starts afterwards. All nonoverlapping vector form patches (*n*×1 sized) are arrayed to produce a matrix from lowpass UDCT sub-band of intermediate image. Results of (**D** ^*r*^)^*†*^ multiplying with the above matrix are the representation coefficients $\boldsymbol {\Gamma }^{r}_{R}$ (differing from double sparse coefficients) of lowpass UDCT sub-band coefficients over the dictionary. They are generally not sparse but easier to handle in our reconstruction approach. The composite regularization reconstruction formulation is solved by using VS and ADMM-2 based on C-SALSA thoughts in an iterative procedure (an updated intermediate image for once iteration), which involves modifying the representation coefficients, UDCT sub-bands coefficients and *k*-space measurements. The proposed method is named as local sparsity enhanced CS MRI(LSECSMRI). Formulations and implementations of the proposed sparsity structure and relevant reconstruction model are described in detail in the following content.

#### Composite sparsity structure

To the best of our knowledge, DWT is not applicable for 2D image sparse representation [55, 56] because of its very limited directions and incapacity to capture geometric regularity along singularities of surfaces. Multi-scale geometric analysis(MGA) methods like contourlet transform [56], nonsubsampled contourlet transform(NSCT) [57], SFLCT, FDCT and DST, etc, conquer the defects of DWT. Contourlet transform lacks shift-invariance though, which causes pseudo-Gibbs phenomena around singularities. The resulting contourlets cannot ensure good frequency localization and exhibit some fuzzy artifacts inevitably with a low redundancy of 4/3. NSCT is overcompletely designed with better frequency selectivity, regularity and fully shift-invariance. But it possesses very high redundancy and large time consumption. SFLCT is a semi-redundant contourlet transform providing flexible redundancy. It only increases redundancy in the lowpass filter *l*_0_(*ω*) to reduce pseudo-Gibbs phenomena, which are mainly induced by down-sample in the lowpass filter. The redundancy can be 2.33 if we don’t down sample *l*_0_(*ω*) and 1.33 if we down sample it by setting *↓*(*d*,*d*)=*↓*(2,2), where *d* is the down-sample parameter that determines the redundancy of contourlet. But SFLCT performs negatively in capturing clear directional features. Similarly, DST provides low redundancy less than or equal to 2. The needle-shaped elements of FDCT allow very high directional sensitivity and anisotropy and are thus very efficient in representing line-like edges. Nevertheless, FDCT possesses too high redundancy, which makes it sub-optimal in sparse representation, either.

UDCT [46] is recently proposed as an innovative implementation of discrete curvelet transform disposing real-valued signals. Utilizing the ideas of FFT-based discrete curvelet transform and filter-bank based contourlet transform, UDCT is designed as a perfect multi-resolution reconstruction filter bank(FB) and executed by FFT algorithm, possessing advantages of the both. The discrete curvelet functions in UDCT are defined by a parameterized family of smooth windowed functions that satisfy two conditions: 1) 2*π* periodic; 2) their squares form a partition of unity and the centers of the curvelet functions at each resolution are positioned on a uniform lattice. Moreover, the lattices of lower scales are subset of those at higher scales, guaranteeing clear parent-children relationship. UDCT can provide flexible instead of fixed number of clear directions at each scale to capture various directional geometrical structures of image accurately. Besides, the forward and inverse transform form a tight and self-dual frame with an acceptable redundancy of 4, allowing the input real-valued images to be perfectly reconstructed. In terms of the implementation of UDCT, once all the curvelet windows are computed, the actual forward and inverse UDCT computations are straightforward. UDCT has asymptotic approximation properties: for image **x** with *C* ^2^ (*C* is a constant) singularities, the best *N*-term approximation **x**_*N*_ (*N* is the number of most important coefficients allowing reconstruction) in the curvelet expansion is given by [12, 55, 58]

(8) $$ \left\|\textbf{x}-\textbf{x}_{N}\right\|^{2}_{2} \leq CN^{-2}\left(\text{logN}\right)^{3} \ \ N\rightarrow{} \infty  $$

This property is known as the optimal sparsity. Therefore, UDCT is considered as the optimal predefined sparse method and is introduced into CS MRI in this paper.

UDCT belongs to predefined sparse priors, which implies that it lacks adaptivity. Explicit dictionary representations in spatial domain gain a higher degree of freedom in the training but sacrifice efficiency of the result. Besides, this kind of explicit dictionary cannot describe hierarchical structures. Incorporating the advantages of UDCT with DL and compensating for the defects of each other, an efficient composite sparsity structure is proposed for local sparsity enhancement. This structure learns adaptive dictionary over lowpass UDCT sub-band coefficients patches. For real (imaginary) part of image **x**, the DL problem is deduced as

(9) $$ \min_{\textbf{D}^{r}, \boldsymbol{\Gamma}^{r}} \sum\limits_{ij}\left\|\textbf{R}_{ij}(\boldsymbol{\Psi} \textbf{x}^{r})_{l}-\textbf{D}^{r}\boldsymbol{\alpha}_{ij}\right\|^{2}_{2} \ \ \text{s.t.} \ \left\| \boldsymbol{\alpha}_{ij}\right\|_{0} \leq T_{0}, \forall i,j  $$

**Fig. 2:**
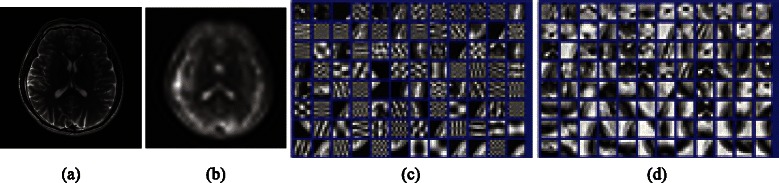
Dictionary training. (**a**) MR T2wBrain_slice_27, (**b**) real part lowpass UDCT sub-band coefficients for DL, (**c**) initial dictionary from 8×8 sized coefficient patches and (**d**) trained dictionary of 64×100. Dictionary atoms are represented using 2 coefficients each

In (9), ***Ψ*** represents UDCT operator. **x** ^*r*^ denotes real (imaginary) part if *r*=1(0) and the subscript *l* denotes lowpass sub-band. The resulting **D** ^*r*^ is thus dictionary for real (imaginary) part. ***Γ*** ^*r*^ is double sparse representation set of {***α***_*ij*_}_*ij*_. The proposed composite sparsity structure has some advantages. Compared with predefined sparse priors, it provides adaptivity and sparser representations for lowpass sub-band coefficients according to the different structural features of lowpass and highpass UDCT sub-bands coefficients. Compared with explicit dictionary, it allows hierarchical sparsity (depending mostly on UDCT) and reduces overfitting and instability in handling noise. Contrast with double sparsity model, it reduces the amount of calculations for not training dictionaries over highpass UDCT sub-bands, which are generally sparse enough. Therefore, the proposed sparse method can fine fit and hierarchically sparsify MR images with important characteristics preserved and avoid wasting time, making a big difference for improved MR image reconstruction performance. In Fig. 2, an example of dictionary trained by using Sparse K-SVD according to the proposed sparsity structure is exhibited. The used image is complex-valued T2-weighted brain image of slice 27 (MR T2wBrain_slice_27 of 256×256 sized). It is acquired from a healthy volunteer at a 3T Siemens Trio Tim MRI scanner, using the T2-weighted turbo spin echo sequence (*T* *R*/*T* *E*=6100/99 ms, 220×220 mm field of view, 3 mm slice thickness) [37]. The training maximum overlapped patches are 8×8 sized and obtained from lowpass UDCT sub-band coefficients after *S*=1 level’s UDCT decomposition for real part of MR T2wBrain_slice_27. One can see that the resulting dictionary is a highly structured matrix, implying several favorable properties.

#### Compressed sensing MRI reconstruction

The proposed reconstruction model and involved reconstruction procedure are demonstrated in this section. It has been proved that composite regularization performs better than either spatial image TV regularization or sparse coefficients *l*_1_ regularization in reconstruction [5, 21, 33]. Besides, the lowpass UDCT sub-band ***α***_*l*_ is the rough approximation of spatial image to be reconstructed and contains large amounts of information, which indicates that TV regularization on lowpass UDCT sub-band coefficients may further improve edge details and suppress noise, promoting the reconstruction quality. According to the different structural features of spatial image, lowpass sub-band ***α***_*l*_ and highpass sub-bands ***α***_*h*_, a new composite regularization reconstruction model is thereby proposed to handle various regularization efficiently in this paper. The reconstruction model can be denoted as

(10) $$\begin{array}{@{}rcl@{}} \widetilde{\boldsymbol{\alpha}}&=&\min_{\boldsymbol{\Gamma}^{r}_{R},\boldsymbol{\alpha}} TV\left(\boldsymbol{\Psi}^{-1}\boldsymbol{\alpha}\right)+\left\|{\boldsymbol{\alpha}_{h}}\right\|_{1}+TV\left(\textbf{D}^{r}\boldsymbol{\Gamma}^{r}_{R}\right)+\left\|\textbf{D}^{r}\boldsymbol{\Gamma}^{r}_{R}\right\|_{1}\\ \textbf{x}&=&\boldsymbol{\Psi}^{-1}\widetilde{\boldsymbol{\alpha}} \ \ \text{s.t.} \ \left\|\textbf{F}_{u} \textbf{x}-\textbf{y}\right\|^{2}_{2} \leq \boldsymbol{\varepsilon} \end{array} $$

where $\boldsymbol {\varepsilon }\in \mathbb {C}^{m}$ is proportional to the noise standard deviation and controls the allowed noise level. The subscript *h* means highpass UDCT sub-bands coefficients. Spatial image TV regularization *T* *V*(***Ψ*** ^−1^ ***α***) and lowpass UDCT sub-band coefficients TV regularization $TV\left (\textbf {D}^{r}\boldsymbol {\Gamma }^{r}_{R}\right)$ regularize image without smoothing the boundaries, guaranteeing clear edge details in the reconstructed result. UDCT sub-bands coefficients *l*_1_ regularization realizes sparsity and automatic selection of the most important characteristics. Undersampled *k*-space data fidelity term makes the reconstruction fitting the measurements. This reconstruction model is effective for embedding efficient composite regularization according to the different structural features of spatial image, lowpass and highpass UDCT sub-bands coefficients. Since TV regularization is capable of maintaining the boundaries of the objects, the reconstructed edge details can be further strengthened by two level of TV regularization. Besides, integrating $\boldsymbol {\Gamma }^{r}_{R}$ modification into lowpass UDCT sub-band coefficients TV and *l*_1_ regularization can guarantee the accuracy of updated $\boldsymbol {\Gamma }^{r}_{R}$, reduce the complexity of the reconstruction model and overfitting significantly.

Basic thought to settle this reconstruction model is based on C-SALSA. And it is slightly different from C-SALSA because the designed composite regularization contains more than one regularization terms. Since parameter ***ε*** is clearly defined and easy to set, the previous AL based methods ignore it and introduce other regularization parameters *λ*_1(2)_. They are thus inefficient [47], such as TVCMRI, FCSA and SALSA, etc. While in the proposed method, the constrained problem (10) is equivalent to an unconstrained one with a discontinuous objective based on the thoughts of C-SALSA

(11) $$ {\begin{aligned} &\min_{\boldsymbol{\Gamma}^{r}_{R},\boldsymbol{\alpha},\textbf{x}} \lambda_{1}\left[TV\left(\boldsymbol{\Psi}^{-1}\boldsymbol{\alpha}\right)+\left\|{\boldsymbol{\alpha}}_{h}\right\|_{1}+TV\left(\textbf{D}^{r}\boldsymbol{\Gamma}^{r}_{R}\right)+\left\|\textbf{D}^{r}\boldsymbol{\Gamma}^{r}_{R}\right\|_{1}\right]\\ &\quad+\lambda_{2}\mathscr{L}_{E(\boldsymbol{\varepsilon},\textbf{I},\textbf{y})}\left(\textbf{F}_{u} \textbf{x}\right) \end{aligned}}  $$

where *λ*_1_ measures the weight of TV and *l*_1_ regularization, *λ*_2_ measures the weight of *k*-space data fidelity and *E*_(***ε***,**I**,**y**)_ is simply a closed ***ε***-radius Euclidean ball centered at **y**. As is shown, (11) can be treated as a special case of *E* *q*.30 in [47] by defining $\Phi \left (\cdot \right)=TV\left (\boldsymbol {\Psi }^{-1}\boldsymbol {\alpha }\right)+\left \|\boldsymbol {\alpha }_{h}\right \|_{1}+TV\left (\textbf {D}^{r}\boldsymbol {\Gamma }^{r}_{R}\right)+\left \|\textbf {D}^{r}\boldsymbol {\Gamma }^{r}_{R}\right \|_{1}$. It has the form of *E* *q*.13 in [47] with *J*=5 number of functions. These functions in (11) are closed, proper and convex (for details, refer to [47]). Minimization problem (11) is allowed to be decoupled into 5 independent and resoluble ones by a particular mapping way. These 5 independent subproblems include spatial image TV regularization *T* *V*(***Ψ*** ^−1^ ***α***), lowpass UDCT coefficients TV regularization $TV\left (\textbf {D}^{r}\boldsymbol {\Gamma }^{r}_{R}\right)$, UDCT sub-bands coefficients *l*_1_ regularization $\left \|\textbf {D}^{r}\boldsymbol {\Gamma }^{r}_{R}\right \|_{1}$ and ∥***α***_*h*_∥_1_ and *k*-space data fidelity $\mathscr {L}_{E(\boldsymbol {\varepsilon },\textbf {I},\textbf {y})}\left (\textbf {F}_{u} \textbf {x}\right)$. Since all the functions in (11) are closed, proper, convex and [**I** ***Ψ*** **F**_*u*_]^*T*^ has full column rank (***Ψ*** itself is a full column rank matrix), convergence of ADMM-2 involving problem (11) is guaranteed according to Theorem1 (Eckstein-Bertsekas, [59]). The solution of (11) is enforced to approach that of (10) via slowly taking *λ*_1(2)_ to very large values by multiplying with a continuous factor *ρ* (a continuation process with initial values as *λ*_10_, *λ*_20_ and *ρ*>1). Introducing ADMM-2 to this particular case requires the definition of the Moreau proximal maps associated with *l*_1_ regularization, TV regularization and $\mathscr {L}_{E(\boldsymbol {\varepsilon }, \textbf {I}, \textbf {y})}$. Since the input of regularizer can be spatial image, UDCT sub-bands coefficients and representation coefficients, ***μ*** is introduced to represent the input of regularization uniformly. The Moreau proximal map function of regularization $\Phi \left (\boldsymbol {\mu }\right):\mathbb {C}^{p}\rightarrow {}\mathbb {C}^{p}$ is denoted as

(12) $$  \boldsymbol{\Theta}_{\Phi}\left(\hat{\boldsymbol{\mu}}\right)=\arg \min_{\boldsymbol{\mu}}\frac{1}{2}\left\|\boldsymbol{\mu}-\hat{\boldsymbol{\mu}}\right\|^{2}_{2}+\Phi\left(\boldsymbol{\mu}\right)  $$

where $\hat {\boldsymbol {\mu }}$ is the result of mapping to ***μ*** according to the mapping relation $\mathbb {C}^{p}\rightarrow {}\mathbb {C}^{p}$. A simple soft threshold method $\boldsymbol {\Theta }_{\lambda _{1}\Phi }(\cdot)=soft(\cdot, \lambda _{1})$ solves the *l*_1_ regularization relevant to ∥***α***_*h*_∥_1_ and $\left \|\textbf {D}^{r}\boldsymbol {\Gamma }^{r}_{R}\right \|_{1}$. The available fast Chambolle’s algorithm [60] handles TV regularization efficiently. Define ***ν***=**F**_*u*_ **x** via VS technique, then the Moreau proximal map of $\mathscr {L}_{E(\boldsymbol {\varepsilon },\textbf {I},\textbf {y})}$ is simply the orthogonal projection of ***ν*** on the closed ***ε***-radius ball centered at **y**, which can be attacked by

(13) $$ \boldsymbol{\Theta}_{\lambda_{2}\mathscr{L}_{E(\boldsymbol{\varepsilon},\textbf{I},\textbf{y})}}\left(\boldsymbol{\nu}\right)=\left\{ \begin{array}{ll} \textbf{y}+\boldsymbol{\varepsilon}\frac{\boldsymbol{\nu}-\textbf{y}}{\left\|\boldsymbol{\nu}-\textbf{y}\right\|_{2}} &\text{if} \left\|\boldsymbol{\nu}-\textbf{y}\right\|_{2} \textgreater \boldsymbol{\varepsilon}\\ \boldsymbol{\nu} &\text{if} \left\|\boldsymbol{\nu}-\textbf{y}\right\|_{2} \leq \boldsymbol{\varepsilon} \end{array} \right.  $$

**Fig. 3:**
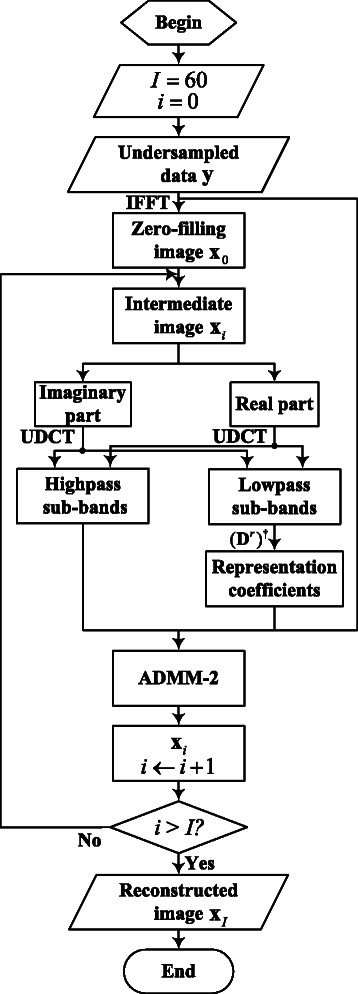
Flowchart for LSECSMRI reconstruction

In ADMM-2, error minimization with respect to ***ν*** aims at fitting measurements of *k*-space. Regularization terms with respect to spatial image, ***α***_*h*_ and $\boldsymbol {\Gamma }^{r}_{R}$ avoid overfitting. The fidelity and regularization terms optimization are implemented alternatively. $\boldsymbol {\Gamma }^{r}_{R}$ is modified from the weighted average between results of $TV\left (\textbf {D}^{r}\boldsymbol {\Gamma }^{r}_{R}\right)$ and $\left \|\textbf {D}^{r}\boldsymbol {\Gamma }^{r}_{R}\right \|_{1}$. Then lowpass UDCT sub-band coefficients are obtained as the weighted average between results of *T* *V*(***Ψ*** ^−1^ ***α***) and $\textbf {D}^{r}\boldsymbol {\Gamma }^{r}_{R}$. The modified highpass UDCT sub-bands coefficients are obtained as the weighted average between results of *T* *V*(***Ψ*** ^−1^ ***α***) and ∥***α***_*h*_∥_1_. Then image in spatial domain is acquired as the result of inverse UDCT to UDCT coefficients. The subproblem with respect to *E*(***ε***,**I**,**y**) can be solved via (13) efficiently. The ultimate reconstructed image **x**_*i*_ (*i* as counter of iterations for ADMM-2) is the result of the reweighted average between the above spatial domain image through regularization penalty and result of (13) for once iteration. Such process reduces reconstruction error and brings about a high-quality reconstructed image efficiently. The flowchart for LSECSMRI reconstruction in Fig. 3 exhibits clearly the reconstruction process based on the proposed sparsity structure.

### Summary of LSECSMRI

In LSECSMRI procedure, ***Ψ*** is uniform discrete curvelet decomposition operator of *S* levels (*s*=1,2,…,*S*) with 2*κ*_*s*_ directions for each level, performing on real and imaginary parts of complex-valued MR data, respectively. The obtained UDCT sub-bands coefficients, representation coefficients of lowpass UDCT sub-band coefficients over **D** ^*r*^ and measurements **y** are sent into the composite regularization reconstruction model for MR image reconstruction. The proposed reconstruction method has some advantages as follows. Firstly, the reconstruction method handles the lowpass sub-band and highpass directional sub-bands coefficients regularization separately with different regularization methods, allowing effective reconstruction. Besides, adding the indicator function of ***ε*** into the objective makes the resulting problem equivalent to the original problem (10) based on the thoughts of C-SALSA. The resulting problem can be decoupled into several subproblems, which are easy to solve with fast convergent algorithms. Additionally, $\boldsymbol {\Gamma }^{r}_{R}$ is modified by using the result of TV and *l*_1_ regularization on lowpass UDCT sub-band coefficients, which reduces the computational complexity and overfitting significantly. Hence, the proposed reconstruction approach provides accurate solution along with rapid convergence speed. Superiorities of LSECSMRI are confirmed experimentally later.

## Results and discussions

### Experimental setup

**Fig. 4:**
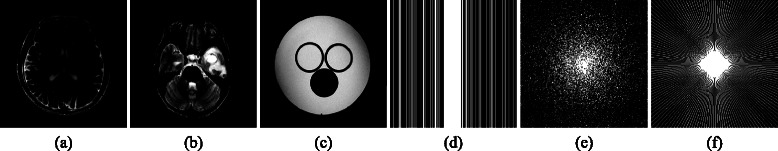
Raw images and sampling schemes. (**a**) T2wBrain_slice_27, (**b**) MBA_T2_slice006, (**c**) water phantom, (**d**) Cartesian sampling, (**e**) 2D variable density random sampling and (**f**) pseudo radial sampling

Experiments are performed under nonuniform sampling schemes at various sampling rates (defined as ratio of $\frac {m}{p}\in \left [0,1\right ]$) in this section. The performance of LSECSMRI is analyzed from 4 different aspects. Images used in the experiments are complex-valued MR T2wBrain_slice_27, complex-valued water phantom acquired at 7T Varian MRI system with spin echo sequence (*T* *R*/*T* *E*=200/100 ms, 80×80 mm field of view, 2 mm slice thickness) [37] and real-valued MBA_T2_slice006 (image courtesy of http://www.med.harvard.edu/AANLIB/home.html). Densities of MR images are normalized to a maximum amplitude of 1 for simulated experiments, via dividing each element value by the maximum module value of image pixels. The mainly applied sampling schemes (binary masks with values of 0 and 1) are Cartesian sampling with random phase encodes http://www.quxiaobo.org/index_publications.html, 2D random sampling http://www.eecs.berkeley.edu/~mlustig/Software.html and pseudo radial sampling [6], etc. Setting of sampling rate depends on numerous experiments. It is appropriate if unobvious visual improvement of reconstructed quality is obtained with sampling rate going on increasing. In simulation, *k*-space measurements are obtained via dot multiplication between Fourier transform coefficients of raw image and sampling matrix at given sampling rate. Raw images and sampling schemes used in experiments are exhibited in Fig. 4. The proposed algorithm is compared with DLMRI, iterative thresholding CS MRI based on more redundant SFLCT(MRSFLCT based CS MRI), LORAKS (rank constraint *r*_*S*_=90 and neighborhood size *R*=4 in *k*-space) and PANO. All experiments are implemented in MATLAB R2011b of a 64-bit Windows 7 operating system with an Intel Xeon E5 CPU at 2.80 GHz and 8 GB memory. Matlab implementations of compared DLMRI, MRSFLCT based CS MRI, LORAKS and PANO are available from the authors’ websites http://mr.usc.edu/code.html, http://www.ifp.illinois.edu/~yoram/Software.html, http://www.quxiaobo.org/index_publications.html. 8 iterations of DL and reconstruction alternation procedure are adopted in DLMRI. Parameters needed in LSECSMRI are manually and empirically set for optimal reconstruction results via numerous of tests. Take UDCT decomposition level *S* and directional sub-bands in each level 2*κ*_*s*_ for example, when they reach certain values, increasing their values doesn’t lead to significant improvement of reconstruction quality but increases computational complexity. Trade-off values are thus obtained by measuring the reconstruction quality and computational complexity. In practice, first commonly set *S*=3 and 2*κ*_*s*_=12, then reduce and increase them to observe the changes of the reconstruction quality. Therefore, to reconstruct images in Fig. 4(a) and (b), UDCT decomposition of *S*=1 and 2*κ*_*s*_=12 acts on intermediate images to obtain 13 coefficient sub-bands, including one lowpass sub-band and 12 highpass directional sub-bands for real and imaginary parts separately. For reconstructing image in Fig. 4(c), UDCT decomposition of *S*=3 and 2*κ*_*s*_=12 is employed. Size of dictionary is 64×100. For T2wBrain_slice_27 reconstruction, initial values of *λ*_1_ and *λ*_2_ are *λ*_10_=*λ*_20_=0.005, continuous factor is *ρ*=1.3. For water phantom reconstruction, *λ*_10_=*λ*_20_=6, continuous factor is *ρ*=1.4. The preset maximum number of ADMM-2 iterations *I*=60 is used as ultimate iteration stop criterion. Numerical metrics of quality assessment for reconstructed images are peak signal-to-noise ratio(PSNR) (in dB), transferred edge information(TEI) [61] and relative *l*_2_ norm error(RLNE) [36].

### Comparison with earlier CS MRI methods

The whole performance of LSECSMRI is tested on raw images in Fig. 4(a)-(c) in this section. The proposed method is compared with DLMRI, MRSFLCT based CS MRI, LORAKS and PANO. Computational time is recorded accordingly. For DLMRI reconstruction, the recorded computational time is that of the first iteration. And the computational time of each iteration increases with iteration number.

**Fig. 5:**
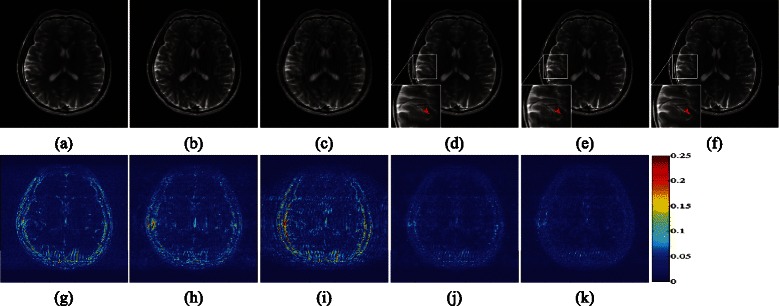
Performance of algorithms under Cartesian undersampling scheme at 35 *%* sampling rate. (**a**)–(**e**) Reconstructed images using DLMRI, MRSFLCT based CS MRI, LORAKS, PANO and LSECSMRI, respectively, (**f**) reconstructed image from fully sampled *k*-space data, (**g**)–(**k**) difference images between fully sampled MR image and images in (**a**)–(**e**) with gray scale of [0,0.25], respectively. PSNRs of them are 31.61, 32.28, 28.53, 36.33 and **3** **7** **.** **4** **0**. TEIs are 0.6109, 0.6306, 0.4886, 0.7529, and **0** **.** **7** **6** **7** **4**. RLNEs are 0.1620, 0.1500, 0.2310, 0.0941 and **0** **.** **0** **8** **3** **2** separately. And computational time is 56.5+80.4sec, 30.5sec, 664.7sec, 296.8sec and 50+492sec, respectively

Using Cartesian undersampling in Fig. 4(d) at 0.35 sampling rate for raw image in Fig. 4(a), DLMRI, MRSFLCT based CS MRI, LORAKS, PANO and LSECSMRI reconstructed results are demonstrated in Fig. 5. Figure 5(a)-(c) indicate clearly that DLMRI, MRSFLCT based CS MRI and LORAKS reconstructed images show severe edge blurring, aliasing and artifacts, implying the poor performance of these methods. While PANO and LSECSMRI reconstructed images exhibit clear edge details and few artifacts. The local regions of reconstructed images in Fig. 5(d)-(f) are scaled up (by a factor of 2) for detailed observations. They clearly show that LSECSMRI performs slightly better in reconstructing clear curve-like details with 1.1dB higher in PSNR, 0.0145 higher in TEI and 0.0109 lower in RLNE compared with PANO (as areas marked in red arrow show). The corresponding difference image in Fig. 5(k) indicates that LSECSMRI reconstructed image obtains the least reconstruction error at the cost of large amounts of time consumption. The computational time is not the tough problem for it can be reduced by using the significant parallel imaging on graphics processing unit(GPU) like the ones in [18, 41, 62], which will be considered in our future work.

**Fig. 6:**
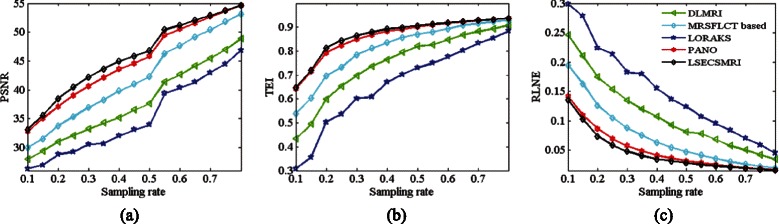
Reconstructed T2wBrain_slice_27 indices versus sampling rates of 2D variable density random sampling schemes. (**a**) PSNR versus sampling rates, (**b**) TEI versus sampling rates and (**c**) RLNE versus sampling rates

Figure 6(a)-(c) presents PSNR, TEI and RLNE indices versus sampling rates of 2D variable density random sampling scheme separately for DLMRI, MRSFLCT based CS MRI, LORAKS, PANO and LSECSMRI reconstructed T2wBrain_slice_27. Figure 6(a)-(c) exhibit that PSNRs and TEIs of DLMRI, MRSFLCT based CS MRI, LORAKS and PANO reconstructed images are lower than those of LSECSMRI reconstructed image overall. When sampling rate is low (between 0.10 and 0.15), LSECSMRI reconstructed image obtain slightly higher PSNR, TEI and lower RLNE compared with that of PANO. While sampling rate gradually increases (sampling rate in the range of 0.15 to 0.45), LSECSMRI reconstructed image achieves considerable higher PSNR, TEI and lower RLNE than PANO reconstructed image. For instance, the PSNR, TEI and RLNE are 40.46dB, 0.8446 and 0.0585 separately for LSECSMRI reconstructed result at 0.25 sampling rate. To obtain comparable results, the sampling rate is approximate 0.30 for PANO, 0.45 for MRSFLCT based method, 0.53 for DLMRI and 0.60 for LORAKS. These indicate that LSECSMRI can use lower sampling rate to obtain comparable reconstruction result as the other four methods under high sampling rates. When sampling rate is high (0.50 and higher), the PSNRs of LSECSMRI reconstructed image are staying at the highest level among the compared five methods. The TEI and RLNE curves of PANO and LSECSMRI reconstructed images are comparable and tend to level off, implying that the reconstructed MR images have already obtain the most information from undersampled data and further more sampled data merely increase the data redundancy and calculations. The overall results demonstrate that LSECSMRI can obtain excellent sparsity structure and reconstruction performance from 2D variable density random undersampling scheme.

**Fig. 7:**
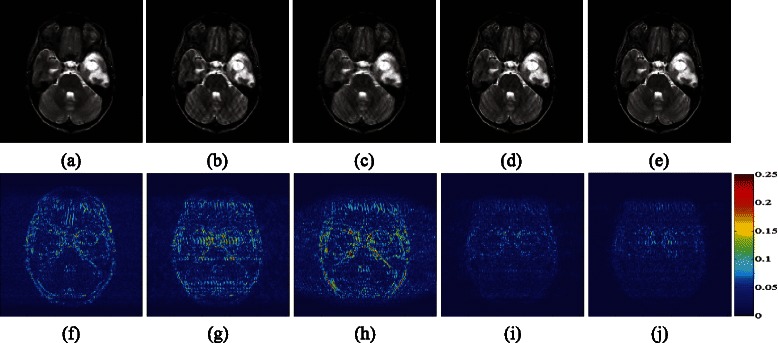
Reconstructed MBA_T2_slice006 under Cartesian undersampling scheme at 0.35 sampling rate. (**a**)–(**e**) Reconstructed images from DLMRI, MRSFLCT based CS MRI, LORAKS, PANO and LSECSMRI, (**f**)–(**j**) difference images between fully sampled MR image and images in (**a**)–(**e**) with gray scale of [0,0.25], respectively. PSNRs of them are 30.95dB, 30.25dB, 28.33dB, 35.21dB and **3** **5** **.** **8** **0**dB. TEIs are 0.5806, 0.5872, 0.5218, 0.7513, and **0** **.** **7** **7** **6** **5**. RLNEs are 0.1152, 0.1249, 0.1558, 0.0705 and **0** **.** **0** **6** **5** **9** separately. And computational time is 62.7+90.5sec, 30.5sec, 711.3sec, 291.2sec and 52.7+514.6sec separately

Figure 7(a)-(e) exhibit reconstructed results from DLMRI, MRSFLCT based CS MRI, LORAKS, PANO and LSECSMRI separately for image in Fig. 4 (b) under Cartesian undersampling scheme at 0.35 sampling rate. The difference images in Fig. 7(f)-(j) show that LSECSMRI reconstructed image possesses the least artifacts and reconstructed error among the compared methods. PSNR of LSECSMRI reconstructed image is 35.80dB, separately 4.85, 5.55, 7.47 and 0.59dB higher than that of DLMRI, MRSFLCT based CS MRI, LORAKS and PANO reconstructed images. RLNE of LSECSMRI reconstructed image is 0.0659, separately 0.0493, 0.0590, 0.0899 and 0.0046 lower than that of DLMRI, MRSFLCT based CS MRI, LORAKS and PANO reconstructed images. These indicate that LSECSMRI can obtain preeminent reconstruction performance among state-of-the-art methods.

**Fig. 8:**
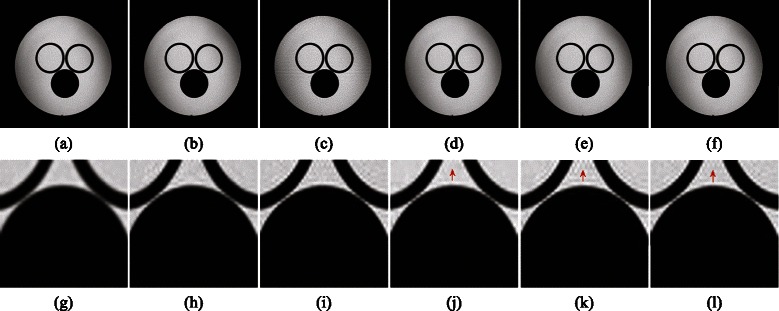
Pseudo radial undersampling at 0.3020 sampling rate for water phantom reconstruction. (**a**)–(**f**) Reconstructed images from DLMRI, MRSFLCT based CS MRI, LORAKS, PANO, LSECSMRI and fully sampled *k*-space data and (**g**)–(**l**) local regions from (**a**)–(**f**). PSNRs for images in (**a**)–(**e**) are 33.14dB, 34.97dB, 31.83dB, 35.76dB and **3** **5** **.** **9** **2**dB. RLNEs for images in (**a**)–(**e**) are 0.0464, 0.0375, 0.0539, 0.0343 and **0** **.** **0** **3** **3** **6**, respectively

Figure 8 exhibits reconstructed results for complex-valued water phantom. *K*-space measurements are obtained via pseudo radial undersampling scheme at 0.3020 sampling rate. Local regions in Fig. 8(g)-(l) (scaled by a factor of 2 to visualize details) exhibit that LSECSMRI shows great superiority in reconstructing clear edges and textures with the least blurring among the compared five methods. While DLMRI reconstructed image introduces severe edge blurring and MRSFLCT based CS MRI reconstructed image exhibits disordered directions.

### Results on noisy data

In this section, the ability in handling noise is demonstrated for the proposed method. Random gaussian white noise with standard deviation 5.1 is added to the original *k*-space data. PSNR, TEI and RLNE are 36.19dB, 0.8208 and 0.0956 for fully sampled T2wBrain_slice_27 and 34.90dB, 0.8813 and 0.0731 for fully sampled MBA_T2_slice006, respectively. In simulation, regularization parameters are manually optimized to obtain maximum PSNRs, TEIs and minimum RLNEs for the compared five methods.

Table 1 and Table 2 show numerical values of PSNRs, TEIs and RLNEs for reconstructed T2wBrain_slice_27 and MBA_T2_slice006 under Cartesian sampling scheme at various sampling rates, respectively. Table 1 and Table 2 exhibit similar change trend. They both show that PSNR and TEI of LSECSMRI reconstructed result are always the highest compared with those of the other four methods reconstructed results when sampling rate is between 0.15 and 0.85, implying superior edge information transfer, low reconstruction error and minimum reconstruction noise of LSECSMRI reconstructed result. As is seen, the PSNR is 34.31dB for LSECSMRI reconstructed T2wBrain_slice_27 at 0.35 sampling rate. To obtain the comparable result, the sampling rate is approximate 0.40 for PANO, 0.75 for LORAKS, 0.60 for MRSFLCT based method and over 0.55 for DLMRI. These indicate that LSECSMRI can use lower sampling rate to obtain comparable reconstructed results as the other four methods at high sampling rates. LORAKS reconstructed result obtains the highest PSNR, TEI and the lowest RLNE when sampling rate reaches 0.95, which is a sign that the significance of sparsity and CS cannot be reflected when sampling rate approaches whole sampling.

**Table 1:** Reconstructed T2wBrain_slice_27 quality indices versus Cartesian sampling rates

Sampling rate	Indices	Methods				
		DLMRI	MRSFLCT based	LORAKS	PANO	LSECSMRI
0.15	PSNR(dB)	24.42	25.16	23.81	27.39	27.73
	TEI	0.1692	0.2939	0.1555	0.3871	0.3879
	RLNE	0.3705	0.3404	0.3975	0.2634	0.2533
0.25	PSNR(dB)	26.68	27.47	25.14	30.88	30.97
	TEI	0.3271	0.4573	0.2599	0.5869	0.5899
	RLNE	0.2858	0.2609	0.3411	0.1761	0.1750
0.35	PSNR(dB)	31.21	31.15	27.83	33.47	34.31
	TEI	0.5927	0.5938	0.4022	0.6719	0.6769
	RLNE	0.1704	0.1708	0.2484	0.1308	0.1188
0.45	PSNR(dB)	31.63	31.91	28.13	34.88	35.58
	TEI	0.6426	0.6629	0.4898	0.7361	0.7330
	RLNE	0.1615	0.1566	0.2418	0.1112	0.1025
0.55	PSNR(dB)	34.20	33.59	30.60	36.06	37.16
	TEI	0.7326	0.6946	0.6140	0.7714	0.7692
	RLNE	0.1202	0.1289	0.1819	0.0970	0.0855
0.65	PSNR(dB)	35.09	35.10	32.22	36.37	38.01
	TEI	0.7666	0.7523	0.6865	0.7902	0.7925
	RLNE	0.1085	0.1084	0.1509	0.0937	0.0775
0.75	PSNR(dB)	35.88	35.46	34.48	36.56	38.92
	TEI	0.7912	0.7681	0.7392	0.8063	0.8105
	RLNE	0.0991	0.1040	0.1164	0.0916	0.0698
0.85	PSNR(dB)	36.29	36.22	37.23	36.44	39.63
	TEI	0.8134	0.8042	0.8057	0.8118	0.8217
	RLNE	0.0945	0.0953	0.0848	0.0929	0.0644
0.95	PSNR(dB)	36.31	36.26	41.86	36.27	40.29
	TEI	0.8196	0.8166	0.8612	0.8183	0.8353
	RLNE	0.0943	0.0949	0.0498	0.0947	0.0596

**Table 2:** Reconstructed MBA_T2_slice006 quality indices versus Cartesian sampling rates

Sampling rate	Indices	Methods				
		DLMRI	MRSFLCT based	LORAKS	PANO	LSECSMRI
0.15	PSNR(dB)	22.86	23.64	22.28	26.22	25.59
	TEI	0.1486	0.2615	0.1418	0.3614	0.3308
	RLNE	0.2924	0.2674	0.3127	0.1985	0.2135
0.25	PSNR(dB)	26.23	26.79	24.55	30.04	30.22
	TEI	0.3427	0.4427	0.2720	0.5530	0.5573
	RLNE	0.1983	0.1860	0.2406	0.1280	0.1253
0.35	PSNR(dB)	30.49	29.27	27.82	32.38	33.26
	TEI	0.5732	0.5585	0.4228	0.6717	0.6703
	RLNE	0.1215	0.1397	0.1647	0.0977	0.0883
0.45	PSNR(dB)	30.93	31.16	27.55	33.75	34.91
	TEI	0.6500	0.6927	0.4961	0.7561	0.7532
	RLNE	0.1155	0.1124	0.1704	0.0835	0.0731
0.55	PSNR(dB)	33.37	33.70	30.30	34.87	36.97
	TEI	0.7467	0.7579	0.6375	0.7994	0.7948
	RLNE	0.0872	0.0840	0.1241	0.0734	0.0576
0.65	PSNR(dB)	34.33	35.00	31.58	35.34	38.14
	TEI	0.8077	0.8296	0.7137	0.8408	0.8329
	RLNE	0.0780	0.0723	0.1072	0.0695	0.0503
0.75	PSNR(dB)	34.67	35.10	33.26	35.25	38.52
	TEI	0.8338	0.8496	0.7708	0.8526	0.8441
	RLNE	0.0751	0.0714	0.0882	0.0702	0.0482
0.85	PSNR(dB)	35.05	35.16	36.73	35.18	39.18
	TEI	0.8621	0.8671	0.8383	0.8639	0.8615
	RLNE	0.0719	0.0710	0.0592	0.0708	0.0447
0.95	PSNR(dB)	34.98	35.01	39.96	34.99	39.76
	TEI	0.8778	0.8769	0.8937	0.8757	0.8766
	RLNE	0.0724	0.0722	0.0408	0.0724	0.0418

### Convergence analysis

Convergence performance of LSECSMRI reconstruction method is analyzed in this section. It is evaluated by MSE decline curve versus successive iterations. Images in Fig. 4(a)-(b) using Cartesian undersampling scheme at 0.35 sampling rate and Fig. 4(c) under pseudo radial undersampling scheme at 0.3020 sampling rate are used for test. LSECSMRI reconstruction is compared with TVCMRI, FCSA, UDCT based C-SALSA reconstruction with *Φ* in *E* *q*.30 in [47] representing *l*_1_ regularization(UDCS_l1) and UDCT based C-SALSA reconstruction with *Φ* in *E* *q*.30 in [47] representing TV regularization(UDCS_TV). The maximum ADMM-2 iteration number is *I*=70. All the parameters are manually optimized for maximum PSNRs, TEIs and minimum RLNEs in reconstruction.

**Fig. 9:**
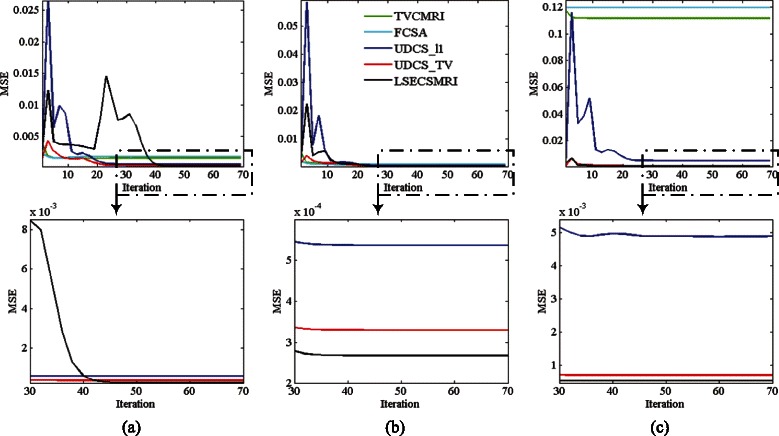
Reconstructed MSE decline versus iteration. (**a**) Reconstructing Fig. 4(a) under Cartesian undersampling at 0.35 sampling rate, (**b**) reconstructing Fig. 4(b) under Cartesian undersampling at 0.35 sampling rate and (**c**) reconstructing Fig. 4(c) under pseudo radial undersampling at 0.3020 sampling rate

Figure 9(a)-(c) exhibit MSE decline curves versus iteration number by using the compared five reconstruction methods for reconstructing images in Fig. 4(a)-(c), respectively. The graphs in the second row show the MSE decline curves in fine scale when iteration number is greater than 30 for UDCS_l1, UDCS_TV and LSECSMRI reconstruction separately. It is concluded from Fig. 9(a)-(c) that LSECSMRI can obtain quite rapid convergence speed with an apparently low MSE, indicating that LSECSMRI reconstruction model is rational and the corresponding reconstruction algorithm is efficient in reconstructing high-quality images.

### Parameters in LSECSMRI

Major parameters in LSECSMRI include UDCT decomposition level (*S*), number of UDCT directional sub-bands for each level, size of coefficient patches (*n*) to train dictionary, number of dictionary atoms (*K*), initial values *λ*_10_ and *λ*_20_ of regularization parameters and the continuous factor *ρ*. Influence of these parameters fluctuation on LSECSMRI reconstruction performance is evaluated by RLNE. The test image is T2wBrain_slice_27. Cartesian undersampling scheme at 0.35 sampling rate is used to undersample *k*-space data of the test image. Experiments indicate that LSECSMRI performs better when *λ*_1_=*λ*_2_ than *λ*_1_≠*λ*_2_. Set the initial parameters values as *S*=1, 2*κ*_*s*_=12, *n*=64, *K*=100, *λ*_10_=*λ*_20_=0.005 and *ρ*=1.3.

**Fig. 10:**
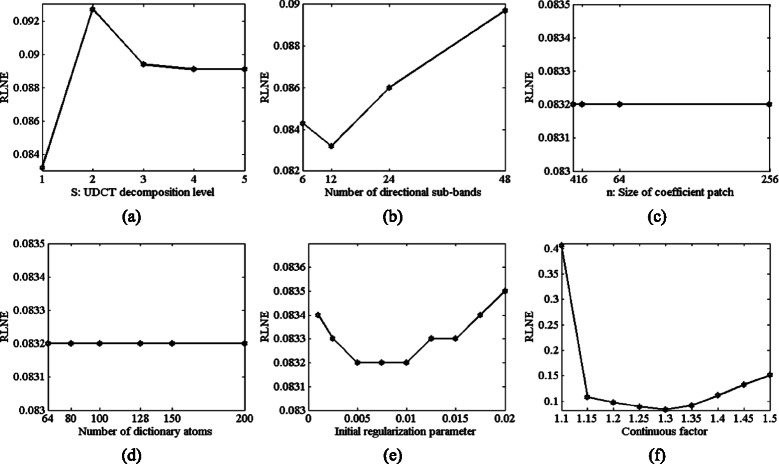
RLNEs versus LSECSMRI parameters for T2wBrain_slice_27 under Cartesian undersampling at 0.35 sampling rate. (**a**) *S* (UDCT decomposition level), (**b**) number of directional sub-bands, (**c**) *n* (patch size), (**d**) number of dictionary atoms (*K*), (**e**) initial regularization parameter *λ*_10_ (or *λ*_20_) and (**f**) continuous factor *ρ* when other parameters are fixed

Increasing *S* within the allowed scope of image size increases the reconstruction error slightly, as is shown in Fig. 10(a). Figure 10(b) illustrates that appropriate number of directional sub-bands gives rise to the least reconstruction error. Reducing and increasing the number both lead to a drop in the reconstructed quality. While size of lowpass UDCT sub-band coefficients patches for DL and number of dictionary atoms seldom affect the reconstruction error, as is shown in Fig. 10(c) and Fig. 10(d). One explanation is that the update of $\boldsymbol {\Gamma }^{r}_{R}$ depends mainly upon the result of TV and *l*_1_ regularization on lowpass UDCT sub-band coefficients. And the TV and *l*_1_ regularization on lowpass UDCT sub-band coefficients are solved by stably convergent algorithms, which guarantees the validity and stability to dictionary size of the reconstructed results. Since parameter *λ*_1(2)_ measures the weight between regularization and data consistency, it makes sense to analyze how the initial regularization parameter *λ*_10_ (or *λ*_20_) and continuous factor *ρ* influence the reconstruction. Figure 10(e) and Fig. 10(f) show influences of *λ*_10_ and *ρ* on the reconstruction performance separately. As is exhibited in Fig. 10(e), increasing *λ*_10_ reduces the reconstruction error when *λ*_10_ is relatively small (*λ*_10_≤0.005 for T2wBrain_slice_27). Whereas too large *λ*_10_ (*λ*_10_>0.01) will increase the reconstruction error. Similar fluctuation trend is obtained for *ρ* and *ρ*=1.3 is suggested for T2wBrain_slice_27 reconstruction.

In short words, a series of experiments indicate that the proposed CS MRI method owns preeminent sparsity structure and reconstruction with rapidly convergent speed, and thereby outperforms current CS MRI techniques in reconstructing high-quality image with clear edge details at a low sampling rate. Experiments on noise, convergence speed and parameters demonstrate its superiority in suppressing noise, convergence and robustness to parameters fluctuation among current CS MRI methods.

## Conclusions and future work

A novel local sparsity enhanced composite sparsity structure is presented, in which UDCT decomposes image to produce structural sparsity and later dictionary is learnt from the lowpass UDCT sub-band coefficients to adaptively sparsify images further. Reconstruction model is then proposed for significant MR images reconstruction established on the proposed sparse representation approach in this paper. Comparing reconstruction performance indicates that the proposed sparsity structure obtains preeminent structural sparsity. The proposed reconstruction model optimizes sparse regularization and constrains measurements fidelity to recover original signal efficiently with a rapidly and stably convergent speed. Experimental results on LSECSMRI agree well with the theoretical analysis, and exhibit superiority in reconstructing highly undersampled MR images under a variety of sampling schemes compared with current CS MRI frameworks. Since the proposed method is simply tested by three MR images in this paper, its universality remains to be investigated. Besides, handling the real and imaginary parts separately doubles the amount of calculations. Further improvements and verifications on the method are subjects of ongoing research and can be made from the following three aspects: (1) test the method on more datasets acquired in real applications; (2) introduce LSECSMRI into practical 3D MRI application by adding the sparse regularization term defined along the temporal axis into the reconstruction model; (3) minimize the modified reconstruction model by using ADMM based methods with partially parallel imaging(PPI) on GPU and faster languages such as C/C++ to speed up the imaging.

## Ethics statement

Brain image of MBA_T2_slice006 was downloaded from Harvard Medical School(http://www.med.harvard.edu/AANLIB/home.html). The rest human images were acquired from healthy subjects under the approval of the Institute Review Board of Xiamen University and written consent was obtained from the participants. The data were analyzed anonymously.
